# Integration of Complete Plasmids Containing *Bont* Genes into Chromosomes of *Clostridium parabotulinum*, *Clostridium sporogenes*, and *Clostridium argentinense*

**DOI:** 10.3390/toxins13070473

**Published:** 2021-07-08

**Authors:** Theresa J. Smith, Renmao Tian, Behzad Imanian, Charles H. D. Williamson, Shannon L. Johnson, Hajnalka E. Daligault, Kristin M. Schill

**Affiliations:** 1 Pathogen and Microbiome Institute, Northern Arizona University, Flagstaff, AZ 86011, USA; terrys2much@comcast.net (T.J.S.); chase.williamson@nau.edu (C.H.D.W.); 2 Institute for Food Safety and Health, Illinois Institute of Technology, Bedford Park, IL 60501, USA; rtian@iit.edu (R.T.); bimanian@iit.edu (B.I.); 3 Food Science and Nutrition, Illinois Institute of Technology, Chicago, IL 60616, USA; 4 Los Alamos National Laboratory, Los Alamos, NM 87545, USA; shannonj@lanl.gov (S.L.J.); hajkis@lanl.gov (H.E.D.); 5 Center for Food Safety and Applied Nutrition, Food and Drug Administration, Bedford Park, IL 60501, USA

**Keywords:** integration, plasmids, botulinum, clostridium, parabotulinum, sporogenes, argentinense

## Abstract

At least 40 toxin subtypes of botulinum neurotoxins (BoNTs), a heterogenous group of bacterial proteins, are produced by seven different clostridial species. A key factor that drives the diversity of neurotoxigenic clostridia is the association of *bont* gene clusters with various genomic locations including plasmids, phages and the chromosome. Analysis of *Clostridium sporogenes* BoNT/B1 strain CDC 1632, *C. argentinense* BoNT/G strain CDC 2741, and *Clostridium parabotulinum* BoNT/B1 strain DFPST0006 genomes revealed *bont* gene clusters within plasmid-like sequences within the chromosome or nested in large contigs, with no evidence of extrachromosomal elements. A nucleotide sequence (255,474 bp) identified in CDC 1632 shared 99.5% identity (88% coverage) with *bont*/*B1*-containing plasmid pNPD7 of *C. sporogenes* CDC 67071; CDC 2741 contig AYSO01000020 (1.1 MB) contained a ~140 kb region which shared 99.99% identity (100% coverage) with plasmid pRSJ17_1 of *C. argentinense* BoNT/G strain 89G; and DFPST0006 contig JACBDK0100002 (573 kb) contained a region that shared 100% identity (99%) coverage with the *bont*/*B1*-containing plasmid pCLD of *C. parabotulinum* Okra. This is the first report of full-length plasmid DNA-carrying complete neurotoxin gene clusters integrated in three distinct neurotoxigenic species: *C. parabotulinum*, *C. sporogenes* and *C. argentinense*.

## 1. Introduction

Botulinum neurotoxins (BoNTs) are extremely potent bacterial proteins produced in a variety of clostridial species. While the mechanism of activity of the toxins and the clinical presentation of botulism is consistent, the proteins themselves differ by as much as 70% in amino acid sequence. Currently, seven serotypes and over 40 toxin subtypes have been described [1].

Botulinum neurotoxin (*bont*) genes have been identified within seven distinct clostridial species [2]. The species designations in this article are based on phylogenetic relationships, not taxonomic entities. Accordingly, *Clostridium parabotulinum*, a previously used species designation for proteolytic clostridia that produce BoNT/A, BoNT/B, or BoNT/F, is used here to describe that particular genospecies. This species is sometimes listed as *C. botulinum* group I. The six additional genospecies are *Clostridium botulinum* (or *C. botulinum* group II), *Clostridium novyi sensu lato* (or *C. botulinum* group III), *Clostridium argentinense*, *Clostridium baratii*, *Clostridium butyricum*, and *Clostridium sporogenes*.

The *bont* genes are positioned in two types of toxin gene clusters ranging in size from approximately 12 to 17 kb that contain both structural genes and, typically, a transcriptional regulator. All gene clusters contain a gene that encodes a non-toxin non-hemagglutinin protein, which is believed to play a role in protecting the toxin molecule as it traverses the digestive system of the host. The two types of gene clusters differ in that one type contains three hemagglutinin genes and the other contains three *orfX* genes of unknown function [3].

The identification of prophage plasmids encoding *bont/C* and *bont/D* in *C. botulinum* group III [4,5] (now known as *C. novyi sensu lato*) initiated numerous research studies aimed at elucidating the genomic location of *bont* genes in other BoNT-producing clostridia. As a result, *bont/G* genes were reported to reside within plasmids [6]. However, *bont/A, bont/B, bont/E*, and *bont/F* genes were historically thought to be chromosomally located [7] due to lack of evidence of extrachromosomal elements prior to the development of genetic tools that are now used to identify them. Over the past two decades, plasmid-borne *bont* gene clusters have been discovered among BoNT/A and BoNT/B strains [8,9,10] BoNT/E strains [11,12], and BoNT/F strains [13,14,15]. For every toxin type, there are now examples of *bont* genes within mobile genetic elements and, with the exception of *bont/C* and *bont/D*, within the chromosome. Moreover, there are examples of strains producing toxin subtypes BoNT/A2, BoNT/A3, BoNT/B1, BoNT/B2, BoNT/E1, BoNT/E3, and BoNT/E10, where identical gene clusters reside within the chromosome and within plasmids.

These extrachromosomal elements tend to be species specific [16]. For example, the plasmids harbored by *C. parabotulinum* show no significant similarity with those of either *C. botulinum* [17] or *C. argentinense* [16], or with the bacteriophage sequences from *C. novyi sensu lato* that contain *bont/C* or *bont/D* genes [18]. However, plasmids found in numerous *C. parabotulinum* strains are homologous to plasmids found in the closely related *C. sporogenes* species.

These *bont* gene-encoding extrachromosomal elements provide a mechanism for the transfer of *bont* gene clusters from toxigenic strains to non-toxigenic bacteria, and it is hypothesized that integration of *bont* genes into the chromosome ensures retention of this feature, as the natural loss of plasmid-borne *bont* genes has been documented [19] and their artificial loss following repeated subculture or genetic disruption in the laboratory has also been demonstrated [20,21]. In contrast, there has been no demonstration of the natural loss of chromosomally located *bont* genes in the literature.

The *bont* gene clusters are integrated into the chromosome via homologous recombination between specific matching DNA sequences within mobile genetic elements and the chromosome. These homologous regions align, the DNA is split between specific nucleotides, and the extrachromosomal DNA sequence that contains the *bont* gene cluster is inserted at the excision site. Sometimes these insertions occur within genes, such as the insertion of *bont/E* genes via *rarA* gene excision [13], the insertion of *bont/F6* gene clusters via *topB* gene excision [22], and the insertion of *bont/F3* and *bont/F4* genes via *pulE* gene excision [14]. In several of these cases, a duplicate intact copy of the split gene is also present. However, with other *bont* gene cluster integrations, the site lies within an intergenic sequence, as illustrated by the integration of the *bont/A2* and *bont/F1* gene clusters [13]. The split genes and/or neighboring genes often encode proteins that are involved in DNA manipulations, including insertion sequence elements, transposases, and resolvases.

The generation of complete, finished genome sequences allows for mapping of *bont* gene cluster locations. Comparisons of identical *bont* gene clusters within plasmids versus those that have integrated onto the chromosome affords us the opportunity to better understand the extent and composition of transferred material and identify surrounding genes or genetic elements that may be involved in the specificity of such transfers. With a few exceptions, the transferred material is limited to DNA sequences of ~17–33 kb containing the *bont* gene cluster and a few surrounding genes [3].

This study describes the rare integration of entire plasmid sequences of ~150–250 kb within the chromosomes of bacteria from three clostridial species (*C. sporogenes*, *C. argentinense*, and *C. parabotulinum*) using homologous recombination processes that are similar to those responsible for *bont* gene cluster integrations.

## 2. Results

### 2.1. Plasmid Integration in C. sporogenes CDC 1632

Currently, 24 *C. sporogenes* strains have been identified in the National Center for Biotechnology Information (NCBI) database that carry *bont/B* genes [20,23,24,25]. Seven of these strains contain *bont/B1* gene clusters, 10 contain *bont/B2* gene clusters, and five contain *bont/B6* gene clusters. Table S1 provides details on these genomes. It is notable that for each of these genomes, including that of CDC 1632 as determined in this study, the putative location of the *bont* genes has been determined to be within plasmids. A BLASTn comparison of the chromosomal nucleotide sequence of CDC 1632 with that of CDC 67071 plasmid, pNPD7 showed 99.5% identity with 88% coverage, indicating that the *bont/B1* gene cluster of CDC 1632 was within a plasmid. Alternatively, using pulsed-field gel electrophoresis (PFGE) followed by Southern blot hybridization with a DNA probe specific to *bont/B* [10], it was determined that the *bont/B* gene cluster of CDC 1632 was located within the chromosome. The seemingly contradictory evidence that the *bont/B1* gene cluster was within a plasmid and also within the chromosome required further investigation. Whole genomic sequencing and assembly of *C. sporogenes* strain CDC 1632 verified that it contained no extrachromosomal elements and that the entire identified plasmid sequence was embedded within the chromosome. The depth of coverage for the reads at the chromosomal plasmid insertion sites was 44X and 69X, which is similar to the average read coverage of 52.6X for that genome (Table 1), providing evidence that this was not a mis-assembly. In addition, multiple programs were used to sequence, assemble, and verify the assembly of the closed and finished CDC 1632 genome. Details are included in the Materials and Methods section. This was the first discovery of a fully integrated plasmid within the chromosome of a neurotoxigenic clostridia.

**Figure 1:**
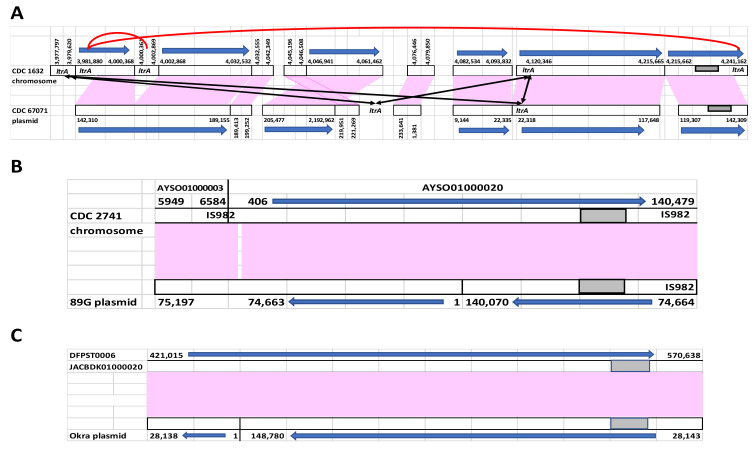
Mapping of nucleotide sequences between *bont* gene-containing plasmids with matching chromosomal sequence within the related strains. Matching regions are shown with pink boxes and *bont* gene cluster positions are designated by gray boxes. The numbers indicate the base pair locations of matching sequences. (**A**) the *bont/B1*-containing plasmid, pNPD7, of *C. sporogenes* BoNT/B1 strain CDC 67071 was found within the chromosome of *C. sporogenes* BoNT/B1 strain CDC 1632, inserted in the same orientation as the chromosome. Two *ltrA* genes positioned prior to and within the inserted material in the CDC 1632 chromosome are identical to the two *ltrA* genes located within the pNPD7 plasmid sequence (black arrows). Two additional *ltrA* genes that flank the CDC 1632 chromosomal sequence and one internal *ltrA* gene are identical in sequence but differ by 5% from the other *ltrA* set (red lines). (**B**) Mapping of *bont/G*-containing plasmid, pRSJ17_1 of *C. argentinense* strain 89G to the chromosome of *C. argentinense* strain CDC 2741 contig AYSO01000020. The plasmid was inserted in the opposite orientation as the chromosome, splitting the plasmid at the IS982 site. An additional intact IS982 element was introduced at the beginning of the inserted sequence beginning at bp 406–1 of contig AYSO01000020 and ending in contig AYSO01000003 (bp 6583–5949). (**C**) Mapping of the *bont/B1*-containing plasmid of *C. parabotulinum* BoNT/B1 strain Okra (pCLD) to the chromosome of *C. parabotulinum* BoNT/B1 strain DFPST0006 (Beans) contig JACBDK01000020. The plasmid was inserted in the opposite orientation as the chromosome.

This plasmid sequence was not inserted into the chromosome of CDC 1632 at the designated origin of reference plasmid pNPD7 but was instead inserted in the same orientation beginning at pNPD7 bp 142,310 (CDC 1632 chromosome position bp 3,983,188) and ending at pNPD7 bp 142,309 (CDC 1632 chromosome position bp 4,238,662) (Figure 1A). While pNPD7 is 235,650 bp in length, the inserted region within the chromosome of CDC 1632 is larger, at 255,474 bp. The inserted region within CDC 1632 showed significant similarity to the pNPD7 plasmid but various insertions and deletions have occurred between these DNA segments (Figure 1A), suggesting this plasmid integration event was not recent.

When *bont* gene clusters are integrated into the chromosome, the insertions are frequently found within a split gene sequence, but sometimes within intergenic sequences. The CDC 1632 plasmid sequence insertion was inserted within an intergenic region, with nearby *ltrA* genes present at the beginning and end of the inserted sequence. The *ltrA* genes encode self-splicing group II intron reverse transcriptase/maturase proteins (IEP) that are a component of Ll,ltrB introns. These introns insert foreign DNA from mobile genetic elements into the chromosome [27]. The *ltrA* protein is multifunctional, mediating RNA splicing, target site recognition, target DNA nicking, insertion of spliced RNA and synthesis of complementary DNA at the insertions site [28]. The Ll.ltrB intron is a component of the TargeTron and ClosTron technologies that are able to generate targeted mutants in *E. coli* and *C. botulinum*, respectively [28]. Their location adjacent to the plasmid insertion site within the chromosome of strain CDC 1632 indicates their involvement in facilitating the chromosomal integration of the pNPD7-like plasmid sequence. Five *ltrA* genes were found in the chromosome of CDC 1632 that were within or flanking the plasmid integration site, and two of these *ltrA* genes were also located within the CDC 67071 pNPD7 plasmid. The *ltrA* genes can be separated into two variants having 5% difference in nucleotide sequence (Table 2). One of these variants was exclusive to the CDC 1632 chromosome and included genes that flanked the plasmid insertion sequence (NPD5_3760; bp 3,981,276–3,983,102 and NPD5_4078; bp 4,239,250–4,241,076), while the other variant (NPD5_3758; bp 3,977,797–3,979,620) matched the two *ltrA* genes within the pNPD7 sequence (NPD7_3844 and NPD7_4079) (Figure 1A).

Two additional *ltrA* genes were located remotely from the plasmid insertion site within the CDC 1632 chromosome and a single *ltrA* gene was found within the chromosome of CDC 67071. A number of phage genes were positioned adjacent to one of these two *ltrA* genes in the CDC 1632 chromosome (NPD5_257; bp 264,722–266,545). The PHASTER program was used to examine the CDC 1632 and CDC 67071 chromosomal genomes for the presence of prophage sequences and complete, intact phage sequences matching that of PHAGE_Clostr_phiCD6356_NC_015626 were discovered adjacent to the *ltrA* gene located at CDC 1632 NPD5_257 and the lone CDC 67071 *ltrA* gene (NPD7_1244) (Table 3), indicating that these *ltrA* genes may be facilitators for the integration of both prophage and plasmid sequences.

### 2.2. Plasmid Integration in C. argentinense Strain CDC 2741 (GM 77/78)

BoNT/G-producing strains are quite rare. Since their discovery in 1969 [32], only two BoNT/G strains have been isolated in Argentina and six in Switzerland [33,34]. While originally considered to be *C. botulinum* strains, in 1988 it was proposed that BoNT/G-producing strains be considered a new species, *C. argentinense*, due to physiological and genetic differences with other *C. botulinum* strains and their similarity with several non-neurotoxigenic clostridia [34].

Evidence for a plasmid location for *bont/G* genes was initially provided by Eklund et al. [35] in 1988 and was confirmed by later studies [6]. Our analysis of the eight *C. argentinense* genomes available in the NCBI database revealed that seven of these genomes, including that of CDC 2741, contained *bont/G* genes, and comparisons with the pRSJ17 plasmid from strain 89G indicated that these *bont/G* genes are universally found within closely related plasmids (98% coverage with 100% identity) (Table S2).

Similar to the genome of CDC 1632, genomic analysis of CDC 2741 provided evidence that the plasmid in this strain was also integrated into the chromosome. The genome of strain CDC 2741 consists of 20 separate contigs (AYSO01000001-AYSO01000020) [16]. One of these contigs, AYSO01000020, was 1.1 Mb in length and contained the *bont/G* gene cluster, suggesting a chromosomal position within the genome. Comparison of CDC 2741 contig AYSO01000020 with reference plasmid pRSJ17_1 of strain 89G revealed the presence of a complete integrated ~140 kb plasmid sequence (100% coverage/100% identity). Read coverage of 156X and 128X were noted for the overlapping reads at the plasmid insertion site, which is similar to the average coverage of 172X for CDC 2741 listed in Table 1. In addition, multiple programs were used to sequence, assemble, and verify the assembly of the CDC 2741 genome.

Alignment of the 89G pRSJ17_1 plasmid sequence with CDC 2741 contig AYSO01000020 revealed that pRSJ17_1 was inserted in reverse orientation within this contig beginning at pRSJ17_1 bp 74,664 (AYSO01000020 bp 140,479) and ending at pRSJ17_1 bp 74,663 (AYSO01000020 bp 406) (Figure 1B). The inserted plasmid sequence began and ended with IS982 elements.

Insertion sequences (IS) are small bacterial transposons containing a transposase, the enzyme that catalyzes DNA movements, flanked by short terminal inverted repeats. IS982 insertion sequences are a class of IS elements that were first discovered in *Lactococcus* spp. and have subsequently been identified in multiple bacterial species [36]. The IS982 element at the beginning of the inserted plasmid sequence was truncated and the other part of this IS982 element was found at the 3′ region of contig AYSO01000003 (bp 6583–5949). In contrast, only one IS982 element was located within plasmid pRSJ17_1. Hence, the insertion of the plasmid sequence in CDC 2741 may have been facilitated through homologous recombination with the participation of these identical IS982 elements. The nucleotide sequence identity shared between plasmid pRSJ17_1 and the plasmid sequence in contig AYSO01000020, and the lack of DNA insertions or deletions, suggests that this was a recent integration event.

### 2.3. Plasmid Integration in C. parabotulinum DFPST0006 (NCTC 7273, Beans)

Genomic sequences representing more than 184 *C. botulinum* (genospecies *C. parabotulinum*) genomes are listed in the NCBI database as *Clostridium botulinum*. Unlike *C. argentinense* and *C. sporogenes*, the majority of *bont* gene clusters in *C. parabotulinum* are found within the chromosome. Plasmid-borne *bont* genes are found in 24 of the *C. parabotulinum* genomes listed in the NCBI database (Table S3). The majority of these plasmids contain more than one *bont* gene cluster (*bont/A1* or *bont/A2* plus *bont/B*; *bont/B5* plus *bont/A4*; and *bont/B5* plus *bont/F2*). The remaining genomes contain solitary *bont/A3*, *bont/B*, or *bont/F5* genes.

The genome of BoNT/B1-producing *C. parabotulinum* strain DFPST0006, also known as NCTC 7273 or the Beans strain, was recently sequenced in 29 contigs. This strain was isolated from a case of foodborne botulism in the U. S. and its toxin was used to produce the WHO International BoNT/B Antitoxin Standard in 1962 in the UK [37]. The DFPST0006 genome is closely related to that of another U.S. isolate, *C. parabotulinum* subtype BoNT/B1 strain Okra. However, they differ in their *bont* gene locations. While the *bont/B1* gene cluster in strain Okra is within a 149 kb plasmid (pCLD), in strain DFPST0006 this entire plasmid sequence, including the toxin gene cluster, was located within a 573 kb contig (JACBDK01000020) containing both chromosomal and plasmid sequence. The matching region between contig JACBDK01000020 and the pCLD plasmid sequence shows 100% identity with 99% coverage. Read coverage of 156X and 128X was noted for the overlapping reads at the plasmid insertion site, which is similar to the average read coverage of 100X for DFPST0006 listed in Table 1. Mapping of plasmid pCLD to contig JACBDK01000002 revealed that, as with the other two examples of complete plasmid integration, the insertion points were not aligned with the assembly-designated plasmid origin. The plasmid sequence was split at reference plasmid pCLD position bp 28,138–28,143 with a six-nucleotide overlap and was inserted in reverse orientation within the chromosome of strain DFPST0006 from JACBDK01000020 bp 421,015 to bp 570,638 (Figure 1C). The region in the chromosome where the split occurred (the plasmid insertion site) was within an intergenic region having no adjacent identified genes or repeat gene sequences, so that no facilitating genes or elements were located. The 100% identity of the Okra pCLD plasmid with the integrated plasmid in DFPST0006, coupled with a lack of insertions, deletions, or inversions, suggests that this was a recent integration event.

## 3. Discussion

It is hypothesized that the acquisition of *bont* gene clusters by non-neurotoxigenic bacteria offers a distinct advantage to the bacteria, as it elevates these organisms from commensal soil inhabitants to pathogens that are capable of fatally intoxicating large numbers of fish, birds, and mammals, leading to better proliferation and spread. The *bont* gene clusters may be located within extrachromosomal plasmids or prophage sequences, or within the chromosome. While the presence of *bont* gene clusters within extrachromosomal elements may be transient due to plasmid or prophage DNA loss, *bont* gene clusters that reside within the chromosome are stably integrated and less subject to elimination. Thus, the integration of *bont* gene clusters into the bacterial chromosome provides a greater stability of this trait, ensuring its continuity in subsequent generations.

The integration of *bont* genes within chromosomes typically involves DNA sequences of 13–33 kb in size that contain the *bont* gene clusters and a few additional genes. Prior to this study, integration of larger sequences, such as 150–250 kb plasmid sequences, was unknown.

The processes that resulted in the insertion of entire plasmid sequences within the chromosome of *C. sporogenes* strain CDC 1632, *C. argentinense* strain CDC 2741, and *C. parabotulinum* strain DFPST0006 (Beans) are similar to those that facilitated the integration of individual *bont* genes or gene clusters into the chromosome. The relative rarity of integration of large extrachromosomal plasmids versus that of smaller *bont* gene clusters may simply be related to their size (140–240 kb versus 17–33 kb), but other as-yet-unknown influences may also be factors.

One unanswered question concerning the integration of these large plasmids is that of reversibility. Do these represent examples of mobile extrachromosomal elements that are forever integrated into chromosomes or do they represent a transitional state that is followed by excision from the chromosome and resumption as an intact mobile genetic element? While the answer to this question is not known, there is evidence that indicates the natural state of the plasmids is extrachromosomal. It is recognized that a major mechanism for the dissemination of virulence traits and/or antibiotic resistance in bacteria is through the transfer of these genes that are located within conjugative plasmids [38]. This is specifically true with both *C. sporogenes* and *C. argentinense*, where the overwhelming location for *bont* genes is within mobile plasmids. Of the 24 sequenced *C. sporogenes* strains that contain *bont* genes, there is evidence that the *bont* genes are within plasmids in 22 of these genomes (the remaining two genomes are too fractured to determine *bont* gene location). One of the genomes contains a plasmid that is integrated into the chromosome. There is evidence that all seven *C. argentinense* genomes contain their *bont* genes within plasmids; one of these contains a plasmid that is integrated into the chromosome. In addition, there is evidence of closely related plasmids containing identical *bont/B2* genes in both *C. parabotulinum* and *C. sporogenes*, demonstrating that cross-species horizontal transfer of these genes likely occurred via conjugation of these extrachromosomal elements.

As cases where these plasmids have integrated into the chromosome are rarely observed, it is not possible to definitively state that their subsequent excision is not possible. However, with two of the chromosomally integrated plasmids in this study, facilitating genes have been identified that are associated with integration. The plasmid insertion in CDC 1632 involved *ltrA* genes, which are an essential component in the ClosTron system. The ClosTron system allows directed mutational analysis through targeted DNA insertions using specific introns. This system ensures stable integration of the mutated gene in the chromosome, suggesting the possibility that the chromosomal integration of the plasmid in CDC 1632 might be irreversible. With the chromosomally integrated plasmid in CDC 2741, IS982 insertion elements appear to be involved in facilitating integration. The IS982 elements that remain at the insertion sites are not identical, showing a sequence identity difference of over 2%. Mutations in insertion elements can disable their ability to facilitate horizontal transfer events, and the differences in sequence identity among these specific IS982 elements may be evidence that they are no longer able to facilitate the excision of plasmid DNA from the chromosome. While the above information suggests that chromosomal integration of these plasmids is irreversible, there is no definitive proof either for or against this theory.

The novel findings recorded here expand our knowledge about the movement of *bont* genes among various clostridial strains and species within extrachromosomal elements, and the processes involved in chromosomal integration of these genes that ensure persistence of neurotoxicity in these lineages.

## 4. Conclusions

Typically, chromosomal integration of *bont* genes involves acquisition of toxin gene cluster sequences from extrachromosomal plasmids via homologous recombination. However, the chromosomal integration of complete plasmids is rarely observed in bacteria, and it has been previously unknown among the BoNT-producing clostridia. Examination of the genomes of three different species of BoNT-producing clostridia revealed the first examples of chromosomal integration of large *bont* gene cluster-containing plasmids, illustrating a novel process for the potential generation of stable lineages of BoNT-producing clostridia. This process was not confined to a single *bont* serotype or bacterial species and utilized a variety of targeting and facilitating genes.

## 5. Materials and Methods

### 5.1. Whole-Genome Sequencing

Total genomic DNA (gDNA) extracted from *C. sporogenes* BoNT/B1-producing strains CDC 1632 and CDC 67071 was sequenced using Illumina [39] and PacBio [40] technologies. Genome assemblies were performed by the Los Alamos National Laboratory (LANL) Genome Science Group. The Illumina data were assembled using Velvet, version 1.2.08 [41], while the PacBio data were assembled using HGAP, version 3 [42]. Consensus sequences from both assemblers were computationally shredded and assembled using parallel Phrap, version SPS-4.24 [43,44]. The resulting assembly was brought to closed and finished status through both manual and computational finishing efforts using Consed [45] and in-house scripts. The assembled genomes were checked for misassemblies using BridgeMapper, a proprietary PacBio software package, and the assembled genome sequence was corrected by mapping Illumina reads (300x) back to the final consensus sequences using Burrows–Wheeler Alignment (BWA) [46], SAMtools [47] and in-house scripts. Annotations were completed at LANL using an automated system utilizing the Ergatis workflow manager [48] and in-house scripts. Detailed sequence information on these genomes is shown in Table 2.

The gDNA of *C. parabotulinum* BoNT/B1 strain DFPST0006 was extracted and prepared as described previously [49] and was sequenced using the Illumina MiSeq instrument. Read quality was assessed using FastQC v0.11.8 (Babraham Bioinformatics—FastQC A Quality Control tool for High Throughput Sequence Data). Paired-end reads were trimmed using trimmomatic v0.39 [50]. Reads were assembled into contigs using SPAdes v3.13.0 [51], a reference-free assembler, with k mer sizes of 21, 33, 55, 77, 99, and 127. The contigs were filtered to remove those with length <500 bp and k mer coverage <5. After quality trimming, paired-end reads were aligned to contigs using Bowtie2 v2.4.1 [52]. Base coverage was calculated using SAMtools.

Whole-genome sequencing of the following genomes has been previously described: *C. argentinense* BoNT/G strain CDC 2741 [16], *C. argentinense* BoNT/G strain 89G [26], and *C. parabotulinum* BoNT/B1 strain Okra [9]. Further information on these genomes is listed in Table 1.

### 5.2. Identification of Bont Genes and Their Genomic Locations

The presence and locations of *bont* gene-containing genetic elements (chromosomal versus plasmid) were identified using a combination of NCBI BLASTn searches against the *bont/B2* gene and plasmid pNPD7 from *C. sporogenes* CDC 67071, the *bont/G* gene and *C. argentinense* 89G plasmid pRSJ17_1, or the *bont/B1* gene and *C. parabotulinum* Okra plasmid pCLD [29]. Searches involved a total of 118 published *C. sporogenes* genomes, eight published *C. argentinense* genomes, and 185 published *C. parabotulinum* genomes, plus additional unpublished genomes in our collections.

### 5.3. Identification of Prophage Sequences

*C. sporogenes* chromosomes were also examined for the presence of prophages and prophage sequences using PHASTER online database searches [30,31].

## Figures and Tables

**Table 1:** Genome sequence statistics for the strains included in this study.

					Average Read Coverage		
Strain	Genomic Element	NCBI Accession	Size (Mb)	Contigs	Illumina	PacBio	454
CDC 1632	chromosome	CP013243	4.39305	1	319X	52.6X	
CDC 67071	chromosome	CP013242	4.11655	1	649.91X	4.34X	
	plasmid pNPD7	CP013241	0.23565	1	664.75X	5.05X	
CDC 2741	chromosome	AYSO0100000 [16]	4.74256	20	171.8X	-	9.9x
89G	chromosome	CP014176 [26]	4.66299	1	104.22X	18.98X	
	plasmid pRSJ17_1	CP014175 [26]	0.14007	1	128.79X	36.39X	
DFPST0006	chromosome	JACBDK0100000	4.06783	29	100.5X		
Okra	chromosome	CP000939 [13]	3.95823	1			
	plasmid pCLD	CP000940 [13]	0.14878	1			

**Table 2:** *ltrA* gene locations. The *ltrA* genes in bold font flank the insertion site of the CDC 67071-like plasmid sequence within the CDC 1632 chromosome. The *ltrA* genes in normal font are found within the integrated plasmid sequence in CDC 1632 and CDC 67071 plasmid pNPD7. The *ltrA* genes in red font are associated with the integration of a prophage sequence in both chromosomes. * Sequence identities are shown as percent identity compared to the CDC 67071 chromosomal *ltrA* sequence NPD7_1244 using BLASTn [29].

CDC1632 Chromosome				CDC 67071 Chromosome	
Locus Tag	Location (bp)	Identity *	Locus Tag	Location (bp)	Identity *
NPD5_257	264,722–266,545	100%	NPD7_1244	1,338,963–1,340,786	100%
NPD5_3598	3,820,672–3,822,498	95%			
**NPD5_3758**	**3,977,797–3,979,620**	**100%**	**CDC 67071 Plasmid**		
**NPD5_3760**	**3,981,276–3,983,102**	**95%**	**Locus tag**	**Location (bp)**	Identity *
NPD5_3788	4,000,956–4,002,782	95%	NPD7_3844	22,924–24,747	100%
NPD5_3940	4,120,953–4,122,775	100%	NPD7_4079	225,791–227,614	100%
**NPD5_4078**	**4,239,250–4,241,076**	**95%**			

**Table 3:** Identification and base pair location of intact prophage sequences in the chromosomes of strains CDC 1632 and CDC 67071 using PHASTER [30,31]. The prophage sequences in bold font are associated with CDC 1632 *ltrA* sequence NPD5_257 and CDC 67071 *ltrA* sequence NPD7_1244.

**DC 1632 Chromosome**			
**Size**	**State**	**Location (bp)**	Phage Name (Closest Match)
**51.9 Kb**	**intact**	**261,289–313,268**	**PHAGE_Clostr_phiCD6356_NC_015262(9)**
50.2 Kb	intact	3,627,037–3,677,326	PHAGE_Clostr_phiCTC2B_NC_030951(8)
**CDC 67071 Chromosome**			
**Size**	**State**	**Location (bp)**	Phage Name (Closest Match)
**46.5 Kb**	**intact**	**1,345,771–1,392,323**	**PHAGE_Clostr_phiCD6356_NC_015262(9)**
**CDC 67071 Plasmid**			
No intact phage present; no incomplete phage associated with *ltrA* present.			

## Data Availability

Publicly available datasets were analyzed in this study. The draft genome of newly sequenced strain DFPST0006 was deposited in the NCBI WGS database under Bioproject PRJNA643587 with an accession number of JACBDK000000000. Table 1 contains NCBI accession information for all genomes examined in this study.

## Supplementary Materials

The following are available online at https://www.mdpi.com/article/10.3390/toxins13070473/s1, Table S1: Characteristics of plasmids containing *bont* gene clusters within *C. sporogenes* strains; Table S2: Characteristics of plasmids containing *bont* gene clusters within *C. argentinense* strains; Table S3: Characteristics of plasmids containing *bont* gene clusters within *C. parabotulinum* strains.
