# Supplementary material for: Integration of Complete Plasmids Containing Bont Genes into Chromosomes of Clostridium parabotulinum, Clostridium sporogenes, and Clostridium argentinense
Source: Toxins (Basel). 2021 Jul 8;13(7):473. doi: 10.3390/toxins13070473 (PMC8310154; doi:10.3390/toxins13070473)
Supplement: Supplementary file 1 [file toxins-13-00473-s001.zip › toxins-1250315 supplementary for conversion final.pdf]

Supplementary Materials: Integration of Complete Plasmids Containing *Bont* Genes into Chromosomes of *Clostridium parabotulinum*, *Clostridium sporogenes*, and *Clostridium argentinense*

Theresa J. Smith, Renmao Tian, Behzad Imanian, Charles H. D. Williamson, Shannon L. Johnson, Hajnalka E. Daligault and Kristin M. Schill

Table S1. Characteristics of plasmids containing *bont* gene clusters within *C. sporogenes* strains. Identified complete plasmids are characterized in the upper table. Genomes where contigs matching >70% of reference plasmid CDC 67071 pNPD7 were identified are listed below. *This genome is listed in the NCBI *Clostridium botulinum* database. **The CDC 1632 plasmid is integrated into the chromosome.

Toxin Type	Strain	Plasmid Size (kb)	Plasmid ID	NCBI Accession #	References
B2	Prevot 594	257.337	pCBH	CP006901	[1]
B6	AM553	266.275	unnamed	LFPK01000006	[2]
B6	AM1195	267.642	pRSJ11_1	CP013700	[2]
B6	AM370	265.469	unnamed	LFPJ01000009, LFPJ01000030	[2]
B2	It 450 *	250.014	p_B2_450	JXSU01000010	[3]
B1	CDC 67071	235.65	pNPD7	CP013241	[4]
B1	CDC 1632	**		CP013243	[5]
B1	2113/01	251.706	unnamed	SWSN01000006	[4]
B1	FT236	251.706	unnamed	SXEL01000007	[4]
B1	1779	251.598	unnamed	SXFJ01000006	[4]

Additional *C. sporogenes* strains having plasmid-borne *bont* genes:

Toxin Type	Strain	Toxin Type	Strain
B2	ATCC 51387	B2	IFR 18/108
B2	Prevot 1662	B2	IFR 18/065
B6	Okayama2011	B2	IFR 18/106
B6	Osaka05	B2	BL 81/18B
B1	R1125/03	B2	2345
B1	R1135/03	B2	Colworth BL246

Table S2. Characteristics of plasmids containing *bont* gene clusters within *C. argentinense* strains. *These genomes were deposited in the NCBI *Clostridium botulinum* database by J. Brunt, A.H.M. Van Vliet, S.C. Stringer, A.T. Carter, and M.W. Peck. **The CDC 2741 plasmid is integrated into the chromosome.

Toxin Type	Strain	Plasmid Size (kb)	Plasmid ID	NCBI Accession #	References
G	89G	140.07	pRSJ17_1	CP014175	[6]
G	GH	138.889	unnamed	SWMY01000026<br>SWMY01000028<br>SWMY01000034<br>SWMY01000037	[4]*
G	113/29	138.863	unnamed	SXCA01000022<br>SXCA01000025	[4]*
G	113/31	139.213	unnamed	SXBZ01000027<br>SXBZ01000033<br>SXBZ01000040	[4]*
G	113/33	138.693	unnamed	SXBY01000032<br>SXBY01000036<br>SXBY01000044<br>SXBY01000059	[4]*
G	CDC 2740<br>(GM 73/78)	140.07	pCAG	AB853998	[7]
G	CDC 2741<br>(GM 77/78)	**	unnamed	AYSO01000020	[1]

Table S3. Characteristics of plasmids containing *bont* gene clusters within *C. parabotulinum* strains. Identified complete plasmids are characterized in the upper table. Genomes where contigs matching >70% of reference plasmid Okra pCLD were identified are listed below. *The DFPST0006 plasmid is integrated into the chromosome.

Toxin Type	Strain	Plasmid Size (kb)	Plasmid ID	NCBI Accession #	References
A2B3	It 87	275.57	p1_A2B3_87	AUZB01000012	[8]
A2B5	CDC_1436	275.99	pCBG	CP006909.1	[1]
A2B6	AM282	266.23	pRSJ10_1	CP013684.1	[5]
A2B7	It 92	260.81	p1_A2B7_92	AUZA01000014	[8]
A2F4F5	Af84	246.12	pCLQ	AOSX01000021.1	[9]
A2F5	BrDura	241.08	pRSJ20_1	CP014152.1	[5]
A3	Loch Maree	266.78	pCLK	CP000963.1	[10]
A3	SU0972	238.81	unnamed	MWIV01000007	this study
B1	Okra	148.78	pCLD	CP000940.1	[10]
B2	It 433	266.69	p1_433B2	AUYZ01000011	[8]
B5A4	657	270.02	pCLJ	CP001081.1	[10]
B5A4	CFSAN034200	270.02	p1_CDC51232	CP031095.1	[11]
B5F2	An436	171.02	unnamed	LFON01000008	[2]
B7	BAC-04-07755	131.32	unnamed	SCKF01000025	this study
B7	CDC69068	117.17	unnamed	POTG01000037, POTG01000039	this study
B7	CDC37498	130.86	unnamed	POTJ01000036	this study
F5	SU0634F	244.78	pRSJ2_3	CP013710.1	[5]
F5	SU0632	243.78	unnamed	MWIY01000007	this study
B1	DFPST0006	*		JACBDK01000020	This study

additional strains having evidence of plasmid location for *bont* genes:

toxin type	strain
B5F2	CDC69057
B5F2	Bf
A6B1	CDC 41370
B5A4	CDC 69043
A2B2	301-13

References:

1. Smith, T.J.; Hill, K.K.; Xie, G.; Foley, B.T.; Williamson, C.H.D.; Foster, J.T.; Johnson, S.L.; Chertkov, O.; Teshima, H.; Gibbons, H.S.; Johnsky, L. A. Genomic sequences of six botulinum neurotoxin-producing strains representing three clostridial species illustrate the mobility and diversity of botulinum neurotoxin genes. *Infect Genet Evol* **2015**, *30*, 102-113, doi:10.1016/j.meegid.2014.12.002.
2. Williamson, C.H.; Sahl, J.W.; Smith, T.J.; Xie, G.; Foley, B.T.; Smith, L.A.; Fernandez, R.A.; Lindstrom, M.; Korkeala, H.; Keim, P.; Foster, J. Comparative genomic analyses reveal broad diversity in botulinum-toxin-producing Clostridia. *BMC Genomics* **2016**, *17*, 180, doi:10.1186/s12864-016-2502-z.
3. Fillo, S.; Giordani, F.; Anselmo, A.; Fortunato, A.; Palozzi, A.M.; De Santis, R.; Ciammaruconi, A.; Spagnolo, F.; Anniballi, F.; Fiore, A.; Auricchio, B. Draft Genome Sequence of Clostridium botulinum B2 450 Strain from Wound Botulism in a Drug User in Italy. *Genome Announc* **2015**, *3*, doi:10.1128/genomeA.00238-15.
4. Brunt, J.; van Vliet, A.H.M.; Carter, A.T.; Stringer, S.C.; Amar, C.; Grant, K.A.; Godbole, G.; Peck, M.W. Diversity of the Genomes and Neurotoxins of Strains of Clostridium botulinum Group I and Clostridium sporogenes Associated with Foodborne, Infant and Wound Botulism. *Toxins (Basel)* **2020**, *12*, doi:10.3390/toxins12090586.
5. Smith, T.J.; Xie, G.; Williamson, C.H.D.; Hill, K.K.; Sahl, J.W.; Keim, P.; Johnson, S.L. Genomic characterization of newly completed genomes of botulinum neurotoxin-producing species from Argentina, Australia and Africa. *Genome Biol Evol* **2020**, doi:10.1093/gbe/evaa043.
6. Zhou, Y.; Sugiyama, H.; Nakano, H.; Johnson, E.A. The genes for the *Clostridium botulinum* type G toxin complex are on a plasmid. *Infect Immun* **1995**, *63*, 2087-2091.
7. Sakaguchi, Y.; Uchiyama, J.; Take, A.; Gotoh, K.; Sakaguchi, M.; Suzuki, T.; Yamamoto, Y.; Hosomi, K.; Kohda, T.; Mukamoto, M.; Kozaki, S. Analysis of a plasmid encoding botulinum neurotoxin type G gene in Clostridium argentinense. *Anaerobe* **2020**, *66*, 102281, doi:10.1016/j.anaerobe.2020.102281.

8. Giordani, F.; Fillo, S.; Anselmo, A.; Palozzi, A.M.; Fortunato, A.; Gentile, B.; Azarnia Tehran, D.; Ciammaruconi, A.; Spagnolo, F.; Pittiglio, V.; Anniballi, F. Genomic characterization of Italian *Clostridium botulinum* group I strains. *Infect Genet Evol* **2015**, *36*, 62-71, doi:10.1016/j.meegid.2015.08.042.
9. Dover, N.; Barash, J.R.; Hill, K.K.; Davenport, K.W.; Teshima, H.; Xie, G.; Arnon, S.S. *Clostridium botulinum* strain Af84 contains three neurotoxin gene clusters: *bont*/A2, *bont*/F4 and *bont*/F5. *PLoS One* **2013**, *8*, e61205, doi:10.1371/journal.pone.0061205.
10. Hill, K.K.; Xie, G.; Foley, B.T.; Smith, T.J.; Munk, A.C.; Bruce, D.; Smith, L.A.; Brettin, T.S.; Detter, J.C. Recombination and insertion events involving the botulinum neurotoxin complex genes in *Clostridium botulinum* types A, B, E and F and *Clostridium butyricum* type E strains. *BMC Biology* **2009**, *7*, 1-18, doi:10.1186/1741-7007-7-66.
11. Gonzalez-Escalona, N.; Haendiges, J.; Miller, J.D.; Sharma, S.K. Closed Genome Sequences of Two Clostridium botulinum Strains Obtained by Nanopore Sequencing. *Microbiol Resour Announc* **2018**, *7*, doi:10.1128/MRA.01075-18.
